# Ferulaldehyde Improves the Effect of Methotrexate in Experimental Arthritis

**DOI:** 10.3390/molecules22111911

**Published:** 2017-11-06

**Authors:** Lukáš Slovák, Karol Švík, Danica Mihalová, Jaroslav Tóth, Szilvia Czigle, Ľudmila Pašková, František Bilka, Katarína Bauerová

**Affiliations:** 1 Institute of Experimental Pharmacology & Toxicology, Slovak Academy of Sciences, Dúbravská Cesta 9, 841 04 Bratislava, Slovakia; karol.svik@savba.sk (K.Š.); danica.mihalova@savba.sk (D.M.); 2 Faculty of Pharmacy, Comenius University in Bratislava, Odbojárov 10, 832 32 Bratislava, Slovakia; jaroslav.toth@uniba.sk (J.T.); czigle@fpharm.uniba.sk (S.C.); paskova@fpharm.uniba.sk (Ľ.P.); bilka@fpharm.uniba.sk (F.B.)

**Keywords:** ferulaldehyde, methotrexate, adjuvant arthritis, combination treatment, rheumatoid arthritis

## Abstract

Methotrexate (MTX) is still the gold standard for treatment of rheumatoid arthritis (RA). The therapeutic efficacy of low-dose of MTX can be increased by its combination with a natural substance, ferulaldehyde (FRA). The aim of this study was to evaluate the effect FRA and MTX administered alone or in combination in adjuvant arthritis. The disease was induced to Lewis male rats by intradermal injection, which contains a suspension of heat-inactivated *Mycobacterium butyricum* in incomplete Freund’s adjuvant. The experiment of 28 days included: healthy animals, arthritic animals, arthritic animals with administration of FRA at the oral daily dose of 15 mg/kg, arthritic animals with administration of MTX at the oral dose of 0.3 mg/kg twice a week, and arthritic animals administered with FRA and MTX. FRA in monotherapy decreased significantly only the level of interleukin-1β (IL-1β) and matrix metalloproteinase-9 in plasma. Combination of FRA and low-dose MTX was more effective than MTX alone when comparing body weight, hind paw volume, arthritic score, plasmatic levels of IL-1β, activity of γ-glutamyl transferase, and relative mRNA expression of IL-1β in the spleen. Therefore, the combination treatment was the most effective. The obtained results are interesting for future possible innovative therapy of patients with RA.

## 1. Introduction

Rheumatoid arthritis (RA) is a chronic inflammatory autoimmune disease. A high-risk genetic background, in combination with epigenetic marks and environmental exposures, leads to a cascade of events inducing synovitis with consequent destructive arthritis, also affecting a variety of extra-articular organs [1,2,3]. Many cytokines are active in the joints of patients with RA and play a fundamental role in the processes that cause inflammation, articular destruction, and comorbidities associated with this disease [4]. Adjuvant arthritis (AA) exhibits clinical and pathological features similar to human RA. Although this model may not reproduce all the features of arthritis in humans, it can help to further the understanding of normal inflammatory and immune responses during RA [5].

The treatment of RA is still unsatisfactory, but several powerful disease-modifying antirheumatic drugs have become available, such as methotrexate (MTX). Even in the current era of biological targeted therapies, MTX remains the initial preferred antirheumatic drug as the gold standard for the treatment of RA. The combination of its perceived efficacy, acceptable safety profile, and low cost, as well as decades of clinical experience, makes MTX the cornerstone of treatment for RA and the anchor drug in combination with various biological agents [6]. If MTX is effective, patients usually receive several years of treatment and therefore knowledge about long-term safety is of major importance. However, MTX administration is limited in some cases due to its toxic manifestations. During long-term administration of MTX, frequent adverse events occur such as nausea, mucous ulceration, cytopenia, serious infections, liver damage, and others. Various studies reported that due to toxicity, discontinuation of MTX in patients with RA ranges from 10–37% [7].

There is thus still a need to develop novel antirheumatic drugs and/or new strategies in RA therapy with MTX. One of the strategies could be combinations of low-dose MTX with natural or synthetic substances, which have been suggested not only to improve the efficacy but also to minimize toxic side effects in RA treatment. The selected substance for combination with MTX should possess predominantly anti-inflammatory and additionally antioxidant properties to effectively influence the inflammation and oxidative stress that are part of the pathogenesis of RA.

In this study, we focused on a natural substance, namely, ferulaldehyde (FRA). Its synonyms are coniferyl aldehyde, trans-coniferyl aldehyde, 4-hydroxy-3-methoxycinnamaldehyde, and others [8]. FRA is a water-soluble end-product of dietary polyphenol degradation [9,10]. Ferulates are natural phenolic phytochemicals present in the peels of fruits and vegetables, as well as the bran of grains, seeds, and leaves of different plant species [11]. The antioxidant activity of FRA [12] and its beneficial effects as an antioxidant [13] have been reported. Furthermore, FRA inhibited lipopolysaccharide (LPS)-induced inducible nitric oxide synthase expression and nitric oxide (NO) synthesis in murine macrophage-like RAW 264.7 cells [14]. FRA also exhibited anti-inflammatory effects in a murine septic shock mode [15] and in vitro studies [16,17].

However, the mechanisms of action and the effect of FRA in preclinical and clinical studies have still not been thoroughly investigated and explained. We thus decided to evaluate the effect of FRA and MTX administered alone or in combination in adjuvant arthritis (AA).

## 2. Results

### 2.1. Effect of FRA and MTX Administered Alone or in Combination on Basic Parameters

The change of body weight (CBW) of arthritic animals showed a decrease during the experiment in comparison to the control group on all days monitored (AA vs. CO, day 14, * *p* < 0.05; days 21 and 28, *** *p* < 0.001; Table 1). MTX treatment led to a significant increase in CBW on day 14 (AA-MTX vs. AA, ^+^ *p* < 0.05; Table 1). Administration of FRA to arthritic animals during all 28 days induced no significant modification of CBW compared to group AA. CBW was higher in the group treated with the combination of FRA and MTX compared with the AA group on days 14, 21 and 28 (31.6%, 51.7% and 28.2%, respectively). The effect of the combination treatment was statistically significant on day 21 (AA-FRA-MTX vs. AA, ^+^ *p* < 0.05; Table 1). Oral administration of the combination FRA and MTX thus seemed to improve the increase of body weight of rats in the AA model.

The change in hind paw volume (HPV) showed an increase in the untreated arthritic group compared to the control group on days 14, 21, and 28 (AA vs. CO, ** *p* < 0.01; days 14 and 21, *** *p* < 0.001, day 28; Table 1). Administration of FRA induced no modification of HPV of the arthritic animals on any day monitored. MTX treatment significantly reduced the observed swelling on days 14 and 21 compared to the AA group (AA-MTX vs. AA, day 14, ^++^ *p* < 0.01; day 21, ^+^ *p* < 0.05; Table 1). The addition of FRA to MTX intensified the effect of MTX administration. The effect was statistically significant on experimental days 14 and 21 (AA-FRA-MTX vs. AA, day 14, ^++^ *p* < 0.01; day 21, ^+++^ *p* < 0.001; Table 1). However, the change in HPV was not significant in the treated group AA-FRA-MTX compared with the AA group at day 28 (39.2%).

FRA and MTX administered alone to arthritic animals during the whole experiment induced no significant modification of arthritic score compared to the AA group. Treatment with the combination FRA and MTX significantly reduced the arthritic score observed on days 14, 21 and 28, compared to the AA group (AA-FRA-MTX vs. AA, day 14, ^+++^ *p* < 0.001; day 21, ^++^ *p* < 0.01; day 28, ^+^ *p* < 0.05; Table 1). Moreover, combination treatment with FRA and MTX decreased the arthritic score more effectively compared to MTX alone on days 14 and 21 (AA-FRA-MTX vs. AA-MTX, ^#^ *p* < 0.05; Table 1).

In summary, the combination of FRA and MTX was generally more effective in improving the basic parameters in adjuvant arthritis than the administration of MTX alone.

### 2.2. Effect of FRA and MTX Administered Alone or in Combination on the Activity of γ-Glutamyl Transferase and Interleukin-1β mRNA Relative Expression in Spleen

**Figure 1:**
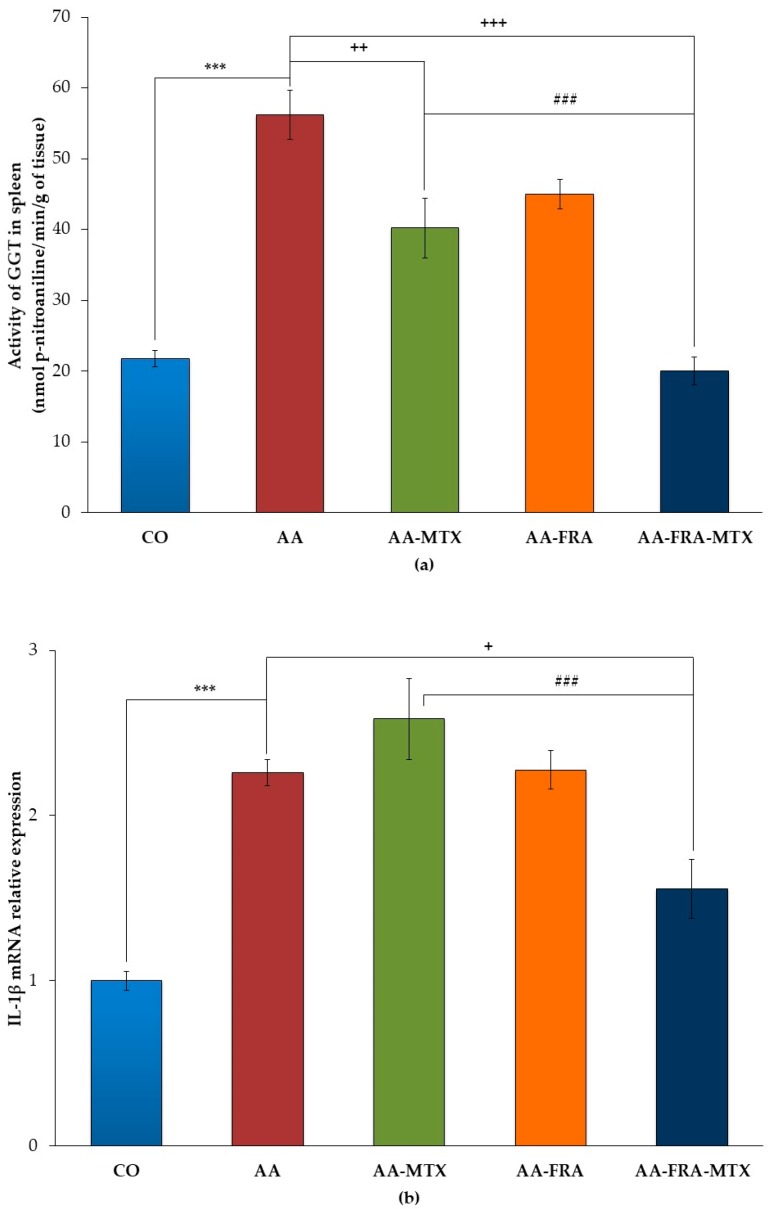
Effect of FRA and MTX administered alone or in combination on the activity of γ-glutamyl transferase (GGT) (**a**) and IL-1β mRNA relative expression (**b**) in spleen on the experimental day 28. CO, control group; AA, adjuvant arthritis group; AA-FRA, adjuvant arthritis group administered ferulaldehyde; AA-MTX, adjuvant arthritis group administered methotrexate; AA-FRA-MTX, adjuvant arthritis group administered ferulaldehyde and methotrexate. The data represent the mean ± SEM; *n* = 7–8. The symbols *, ^+^ and ^#^ show significant difference: *** *p* < 0.001 vs. CO; ^+^ *p* < 0.05 vs. AA; ^++^ *p* < 0.01 vs. AA; ^+++^ *p* < 0.001 vs. AA; ^###^ *p* < 0.001 vs. AA-MTX.

Arthritis increased the activity of γ-glutamyl transferase (GGT) in the spleen of the AA group, which was found to be 2.58 times higher than in the group of healthy animals on day 28 (AA vs. CO, *** *p* < 0.001; Figure 1a). MTX administration led to a significant decrease of GGT activity at the end of the experiment (AA-MTX vs. AA, ^++^ *p* < 0.01; Figure 1a). Although FRA administered alone was without effect on this parameter, the combination treatment restored the value of GGT activity of healthy animals (AA-FRA-MTX vs. AA, ^+++^ *p* < 0.001; Figure 1a). The combination treatment studied was more effective in decreasing of GGT compared to MTX administered alone (AA-FRA-MTX vs. AA-MTX, ^###^ *p* < 0.001; Figure 1a).

An increase of mRNA expression was observed for interleukin-1β (IL-1β) in the spleen of arthritic animals on day 28 (AA vs. CO, *** *p* < 0.001; Figure 1b). FRA and MTX administered alone did not result in any effect on IL-1β mRNA relative expression. The combination treatment studied was the most effective in reducing this parameter in the spleen on day 28 compared to the AA group (AA-FRA-MTX vs. AA, ^+^ *p* < 0.05; Figure 1b) as well as the AA-MTX group (AA-FRA-MTX vs. AA-MTX, ^###^ *p* < 0.001; Figure 1b).

Generally, the effect of FRA and MTX administered in combination had a better effect on parameters in the spleen than had MTX administration alone.

### 2.3. Effect of FRA and MTX Administered Alone or in Combination on the Levels of Interleukin-1β, Interleukin-17A, and Matrix Metalloproteinase-9 in Plasma

**Figure 2:**
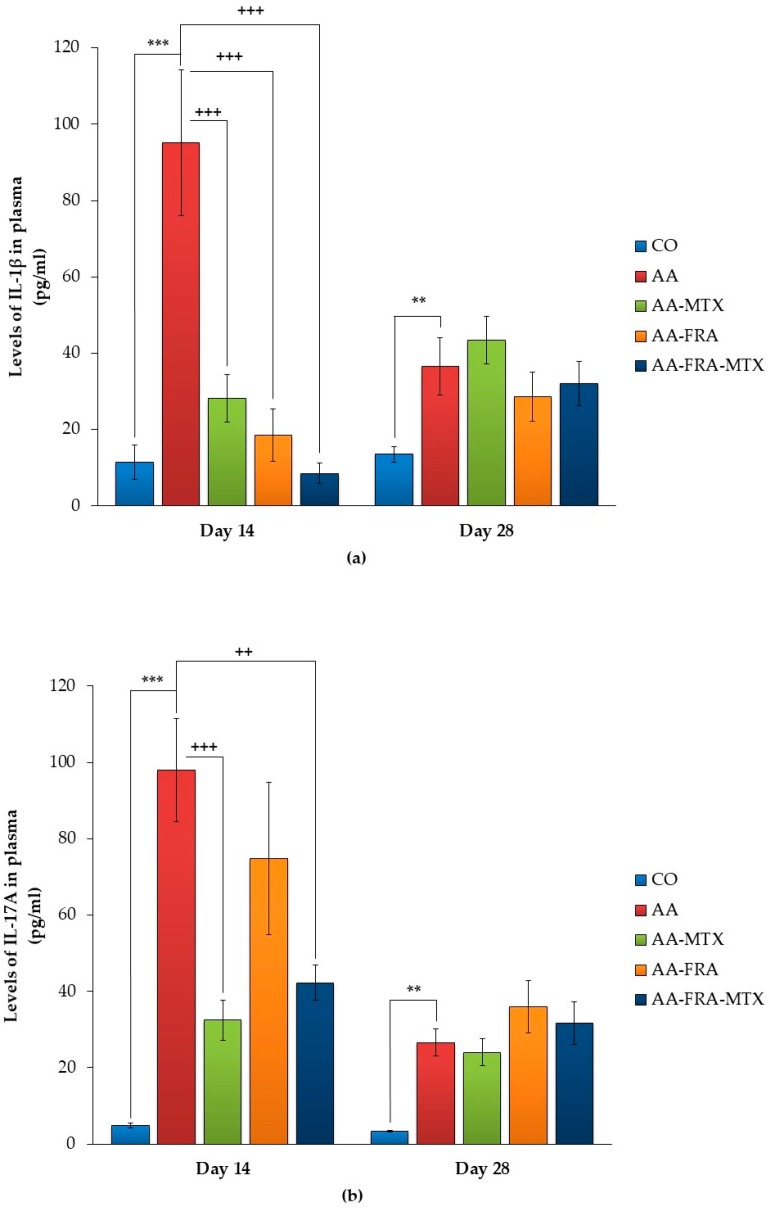

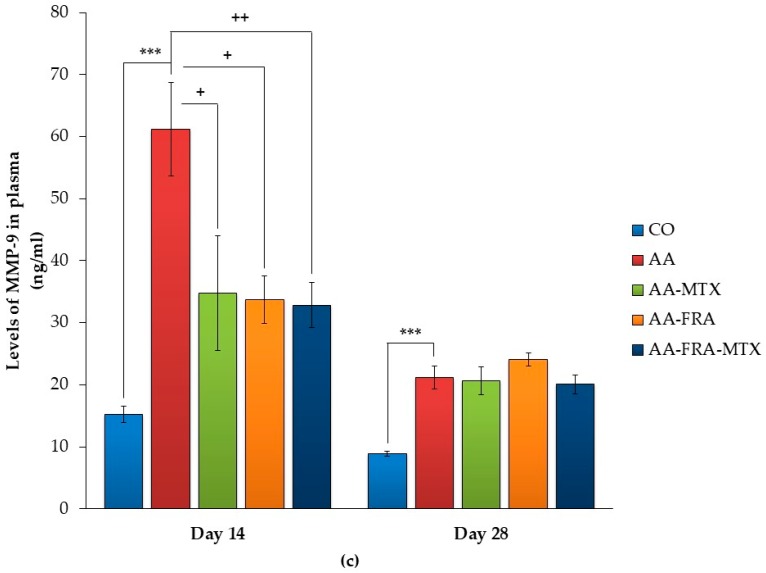
Effect of FRA and MTX administered alone or in combination on the levels of IL-1β (**a**), IL-17A (**b**), and MMP-9 (**c**) in plasma on experimental days 14 and 28. CO, control group; AA, adjuvant arthritis group; AA-FRA, adjuvant arthritis group administered ferulaldehyde; AA-MTX, adjuvant arthritis group administered methotrexate; AA-FRA-MTX, adjuvant arthritis group administered ferulaldehyde and methotrexate. The data represent the mean ± SEM; *n* = 7–8. The symbols *, ^+^ and ^#^ show significant difference: ** *p* < 0.01 vs. CO; *** *p* < 0.001 vs. CO; ^+^ *p* < 0.05 vs. AA; ^++^ *p* < 0.01 vs. AA; ^+++^ *p* < 0.001 vs. AA.

Figure 2a–c show that the levels of IL-1β, interleukin-17A (IL-17A), and metalloproteinase-9 (MMP-9) dramatically decreased in plasma on day 28 compared to day 14 in the AA group of animals. Also, due this reason, we did not observe an effect of FRA and MTX administered alone or in combination on all parameters in plasma on day 28. Thus, the following description of results are focused on day 14.

Plasmatic levels of IL-1β were significantly increased in arthritic animals compared to the control group (AA vs. CO, *** *p* < 0.001; Figure 2a). FRA and MTX administered alone were effective in significantly reducing the IL-1β levels (AA-FRA, AA-MTX vs. AA, ^+++^ *p* < 0.001; Figure 2a). The studied combination treatment was the most effective in reducing the level of this interleukin in plasma on day 14 compared to AA (AA-FRA-MTX vs. AA, ^+++^ *p* < 0.001; Figure 2a).

The level of IL-17A in the AA group was increased approximately 20 times on day 14 when compared to a group of control animals (AA vs. CO, *** *p* < 0.001; Figure 2b). After 14 days of administration of FRA, no significant change was observed in the level of IL-17A. MTX alone significantly decreased the levels of this cytokine in plasma (AA-MTX vs. AA, ^+++^ *p* < 0.001; Figure 2b). Combination treatment significantly reduced the plasmatic levels of IL-17A compared to the untreated group of arthritic animals (AA-FRA-MTX vs. AA, ^++^ *p* < 0.01; Figure 2b).

Further, arthritis significantly increased MMP-9 in plasma on day 14 (AA vs. CO, *** *p* < 0.001; Figure 2c). In the groups of animals treated with FRA and MTX administered alone or in combination, the levels of MMP-9 were significantly reduced on day 14 (AA-FRA, AA-MTX vs. AA, ^+^ *p* < 0.05; AA-FRA-MTX vs. AA, ^++^ *p* < 0.01; Figure 2c).

The combination treatment was effective in reducing the levels of all parameters measured in plasma on day 14, and was the only treatment able to return the levels of IL-1β to the control value of healthy animals.

## 3. Discussion

Adjuvant arthritis (AA) is one of the animal models widely used in studying the mechanisms of RA pathogenesis as well as for preclinical testing of potential antirheumatic drugs and new strategies in therapy for this disease. MTX is a very important drug for monotherapy and combination therapy of RA with other synthetic and biological disease-modifying antirheumatic drugs. However, patients with RA treated with this drug may experience one or more side effects. In some cases, manifestations of MTX toxicity are the reason for discontinuation of the therapy. Therefore, there is a need to develop new strategies in RA therapy with MTX. One possibility is a combination treatment of low-dose of MTX with a synthetic or natural substance, which could increase the efficacy of MTX without increasing its toxicity. Our studies, as well as those of other authors, suggest that the efficacy of low doses of MTX may be increased in this combination [18,19,20,21]. In the present study, we evaluated the anti-arthritic and anti-inflammatory effect of FRA and MTX administered alone or in combination in AA.

In the AA model, chronic systemic inflammation is manifested in increased arthritic score and hind paw volume, as well as in body weight loss. These parameters start to develop approximately 14 days after single intradermal injection of a suspension of heat-inactivated *Mycobacterium butyricum* in incomplete Freund’s adjuvant at the base of the tail. The largest changes in basic parameters were observed on day 21 in the AA group of animals compared to healthy rats, similar to our previous results [22]. Low-dose of MTX had an effect only in the acute phase of AA on HPV and CBW. FRA alone did not exert an effect on the basic parameters measured, though in combination treatment it improved the effect of MTX in correcting CBW, HPV, and especially the arthritic score. In our previous study, the combination of N-feruloylserotonin and MTX also showed the anti-arthritic (hind paw volume and arthritic score) effect [18]. The present results indicate that combination treatment may decrease the signs and symptoms, and thus the severity of the disease. The combination of FRA and MTX appears to be a good candidate for affecting the clinical parameters in patients with RA.

AA is not only an experimental model of polyarthritis, but it also induces pathological changes in a variety of other tissues, particularly useful in splenomegaly [5]. We therefore measured the GGT activity and relative mRNA expression of IL-1β in the spleen, which informed us about local effects of FRA and MTX administered alone and in combination. GGT, as a non-specific marker of inflammation and oxidative stress, is an important component of inflammatory processes since its activity is closely associated with the overall antioxidant status of the organism. FRA alone did not influence the GGT activity on day 28. In the groups of animals treated with MTX, this parameter was significantly decreased. The studied combination treatment was the most effective in reducing the GGT activity in the spleen on the day monitored. Comparison of the MTX group of arthritic animals and the group of rats treated with the combination of MTX and FRA showed a significant increase in the therapeutic efficacy of the combination tested on day 28.

An increase of mRNA expression of IL-1β was observed in the spleen of arthritic rats in comparison with healthy animals, a finding similar to our previous results [22], where we determined the expression of this gene in spleen of the AA model in the rat for the first time. Treatment with low-dose MTX did not significantly affect the level of IL-1β mRNA in accordance with other studies [23,24]. Neither did the administration of FRA to arthritic animals lead to a significant decrease of IL-1β mRNA. However, combination therapy using both MTX and FRA resulted in significant attenuation of arthritis-increased IL-1β mRNA expression in rat spleen.

The influence of the basic AA parameters is associated with significantly increased levels of immunological mediators in plasma (IL-1β, IL-17 and MMP-9), which were detected on day 14. All immunological parameters were analyzed on days 14 and 28. IL-1β is one of the key interleukins that induces the expression of tumor necrosis factor-α (TNF-α), IL-6, and other pro-inflammatory cytokines [25] and acts as a mediator in synovial inflammation and pannus formation [26]. It seems that in most animal models, IL-1β plays a key role in the early stage of adjuvant arthritis [27]. According to Reference [28], this cytokine is important in the degradation of joint cartilage by stimulating synovial fibroblasts and chondrocytes, which produce matrix metalloproteinases (MMPs), cathepsins, and other protein enzymes. In this experiment, we found a significantly higher level of IL-1β on day 14 in the AA group compared to the control group. FRA administered separately significantly decreased plasma levels of IL-1β in rats with AA. This agrees with the results of other authors, where FRA also attenuated the increase in serum levels of TNF-α and IL-1β in the model of inflammation induced by lipopolysaccharide [15]. The combination of FRA and MTX was the most effective in reducing the plasmatic levels of this cytokine.

There is much evidence that IL-17 contributes to inflammation in RA pathogenesis [29]. This interleukin is also detectable in adjuvant arthritis, and its plasma levels are increased in both the acute and chronic phases of the disease. Signs of the onset of AA are followed by an acute intra-articular IL-17 elevation, what probably means that IL-17 is more involved in the progression of the disease than in its induction [30,31]. The relationship between IL-17 levels and other pro-inflammatory cytokines and chemokines is shown in Reference [32]. This author found that decreasing IL-17 by polyclonal rabbit antibodies directed against murine recombinant IL-17 acts against bone erosion and inhibits the expression of pro-inflammatory cytokines (IL-1, TNF-α, and RANKL) and chemokines in the synovium. In our study, MTX and its combination with FRA significantly decreased the level of this interleukin on day 14. The effect of MTX can be explained by its ability to inhibit the production of several pro-inflammatory cytokines: IL-4, IL-6, IL-13, IL-17, TNF-α, interferon gamma, and granulocyte-macrophage colony-stimulating factor [33] in AA and RA. However, FRA had no significant effect on IL-17A, and probably in combination only MTX was responsible for the therapeutic effect.

In the spectrum of inflammatory mediators involved in joint destruction processes during arthritis, matrix metalloproteinases (MMPs) are also important. MMPs are produced by macrophages and synovial fibroblasts [34]. MMP-9 is of particular importance in the degradation of fibrillary collagen types I, II [35] and aggrecan [36]. FRA and MTX administered alone or in combination were able to significantly reduce plasmatic MMP-9 in rats with AA on day 14. MMP-9 is affected by the nuclear factor kappa-light-chain-enhancer of activated B cells (NF-κB) signaling [37]. It was shown that MTX correlated with the inhibition of IκBα degradation and the suppression of its phosphorylation. As a result, NF-κB stays in the cytoplasm and the NF-κB signaling pathway is not activated [33]. FRA diminished MAPK activation, thereby inhibiting NF-κB activation [16]. The decrease of the MMP-9 level by combination is probably due to the inhibition of NF-κB activation by MTX and FRA, as mentioned above.

To improve the anti-arthritic effect of MTX, we selected FRA for combination treatment with MTX, because of its anti-inflammatory properties. In a study with RAW 264.7 macrophage cells, the authors investigated the role of mitogen-activated protein kinases (MAPKs) and MKP-1 activation in the regulation of an early inflammatory response. Macrophages were treated with LPS and FRA inhibited an early inflammatory response. LPS-induced reactive oxygen species (ROS) and nitrogen species formations were reduced by FRA in a concentration-dependent manner, and FRA protected mitochondria against LPS-induced rapid and massive membrane depolarization. LPS induced the early suppression of MKP-1, which was accompanied by the activation of JNK, ERK, and p38 MAPK. By reversing LPS-induced early suppression of MKP-1, FRA diminished MAPK activation, thereby inhibiting NF-κB activation, mitochondrial depolarization, and ROS production. FRA exerts its early anti-inflammatory effect by preserving the mitochondrial membrane integrity and shifting the expression of MKP-1 forward in time in macrophages [16]. Also, MTX exhibits anti-inflammatory activity on the monocyte/macrophage system. One study revealed an MTX-induced downregulation of FcγRI and FcγRIIA expression levels on monocytes in MTX-treated patients, which may thus prevent the activation of monocytes/macrophages via immunocomplexes. The decrease in FcγRIIIA expression levels on monocytes was less marked. In addition, the percentage decrease in FcγRI expression correlated with the decrease in C-reactive protein and well-being [33]. The combination treatment of MTX and FRA probably potentiates their inhibitory activity on macrophages. This effect might be important for the anti-inflammatory mechanism of the combination treatment studied.

Finally, this is the first report in literature evaluating the combination of FRA and low-dose MTX in AA. The obtained results demonstrated that FRA administered alone had a significant effect only on IL-1β and MMP-9 in plasma. However, FRA improved the effect of MTX in combination treatment, especially on basic parameters, parameters assessed in the spleen, and plasmatic IL-1β levels. Thus, the FRA and MTX combination has potential for therapeutic use in chronic inflammatory diseases such as RA. For future preclinical studies, it would be interesting to evaluate the efficacy of FRA and its combination with MTX in others animal models of RA, such as collagen-induced arthritis, to confirm their effectivity and to gain a better understanding their mechanisms of action.

## 4. Materials and Methods

### 4.1. Biological Part of the Experiment

#### 4.1.1. Animals

Male Lewis rats were obtained from the Breeding Farm Dobra Voda (Slovakia) and housed five per cage under standard conditions with food and water ad libitum and a 12-hour-light/12-hour-dark cycle. The experimental protocol was approved by the Ethics Committee of the Institute of Experimental Pharmacology and Toxicology (3144/16-221/3) and by the Slovak State Veterinary and Food Administration in accordance with the European Convention for the Protection of Vertebrate Animals Used for Experimental and Other Scientific Purposes, and was also in accordance with Slovak legislation.

#### 4.1.2. Induction of Adjuvant Arthritis

Adjuvant arthritis (AA) was induced to male Lewis rats weighing 160–180 g by a single intradermal injection of 0.1 mL suspension of heat-inactivated *Mycobacterium butyricum* (Difco Laboratories, Detroit, MI, USA) in incomplete Freund’s adjuvant at the base of the tail.

#### 4.1.3. Experimental Design and Treatments

Rats were randomized into five groups in one experiment: a control group of animals (CO); a group of animals with adjuvant arthritis (AA); a group of animals with AA, which was administered 15 mg/kg FRA daily (AA-FRA); a group of animals with AA, which was administered 0.3 mg/kg MTX twice a week (AA-MTX); and a group of animals with AA, which was administered MTX and FRA in the same doses and regimens as in monotherapy (AA-FRA-MTX). In each group, seven to eight animals were used. The substance and the drug were administered orally during the whole experiment (28 days). Body weight of rats was measured regularly to calculate the precise application doses. Methotrexat^®^ (EBEWE sol inj. 20 mg/1 mL) was diluted with redistilled water. Ferulaldehyde (Sigma-Aldrich) (Darmstadt, Germany) was partially dissolved in redistilled water. On day 14, blood samples were taken from the retro-orbital plexus under Zoletil^®^/xylazine anesthesia. On day 28, the animals were sacrificed under the same anesthesia and blood for plasma preparations was withdrawn along with the spleen from each rat. All samples were stored at −80 °C until biochemical analysis.

### 4.2. Experimental Assays

#### 4.2.1. Basic Parameters

##### Change of Body Weight

Change of body weight (CBW; g) was measured on days 1, 14, 21 and 28. CBW was calculated as the difference of the body mass measured on days 14, 21 and 28 to the body weight measured at the beginning of the experiment (day 1).

##### Change of Hind Paw Volume

The hind paw volume (HPV, %) increase was calculated as the percentage of increase in the HPV on a given experimental day relative to the HPV at the beginning of the experiment. This parameter was recorded on days 1, 14, 21 and 28 with the use of an electronic water plethysmometer (UGO BASILE, Comerio-Varese, Italy).

##### Arthritic Score

The arthritic score was measured as the total score of HPV (ml, max. points 8) + paw diameter of forelimb (mm, max. points 5) + diameter of scab in the site of *Mycobacterium butyricum* application, measured in parallel to the spinal column (mm, max. points 8) for each animal on all experimental days monitored [18].

#### 4.2.2. Parameters Assessed in Tissue

##### Measurement of Activity of γ-Glutamyltransferase in Spleen

The non-specific parameter of inflammation and oxidative stress—γ-glutamyltransferase (GGT)—was determined in the spleen at the end of the experiment, on day 28. The activity of GGT was measured by the method outlined in Reference [38], which was modified according to Reference [39]. Samples were homogenized in buffer 1:9 (*w*/*v*) (composition: 2 mM NaH_2_HPO_4_, 20 mM Na_2_PO_4_, 15 mM EDTA, 68 mM NaCl, pH 8.1) by Ultra Turrax TP 18/10 Janke & Kunkel (Saufen, Germany) for 1 min at 0 °C. Substrates (8.7 mM γ-glutamyl-4-nitroanilide (γ-GPN); 44 mM methionine) were dissolved in 65% isopropyl alcohol with final concentrations in reaction mixture: 2.5 mM γ-GPN; 12.6 mM methionine. After incubation for 60 min at 37 °C, the reaction was stopped by adding 2.3 mL of cold methanol and the tubes were centrifuged for 20 min at 5000 rpm. Absorbance of supernatant was measured by spectrophotometer Specord 40 (Jena, Germany) in a 0.5-cm cuvette at 406 nm. Reaction mixtures in the absence of either the substrate or acceptor were used as reference samples.

##### Total RNA Isolation and Quantitative RT-PCR

Total RNA was extracted from the rat spleen using RNAzol RT (Sigma-Aldrich) and reverse transcribed using the PrimeScript RT Reagent Kit (Takara, Mountain View, CA, USA) following the protocols of the manufacturers. Amplification and detection of cDNA of reference and target genes were performed using HOT FIREPol EvaGreenR qPCR Mix Plus (ROX) (Solis Biodyne, Tartu, Estonia) on a 7300 Real-Time PCR System (Applied Biosystems, Foster City, CA, USA). Relative mRNA expressions of IL-1β were analyzed using the ΔΔCt value method. β-actin was used as a reference gene. PCR products were evaluated by melting curve analysis to confirm the specific amplification. The sequences of the primers were designed and checked using Primer3 and Oligo Analyzer 1.0.3 (Table 2).

#### 4.2.3. Parameters Assessed in Plasma

##### Measurement of Interleukin 1β, Interleukin 17A, and Matrix Metalloproteinase 9 in Plasma

For the determination of interleukin 17A (IL-17A) concentrations in plasma, an ELISA kit from eBioscience^®^ (Waltham, MA, USA) was used. For the determination of plasmatic concentrations of interleukin 1β (IL-1β) and matrix metalloproteinase 9 (MMP-9), an ELISA kit from R&D Systems Quantikine^®^ (Minneapolis, MN, USA) was used. The assay procedures were applied as described in the product manuals. The results were calculated from the standard calibration curves on internal standards.

### 4.3. Statistical Analyses

Mean and SEM values were calculated for each parameter in each group (seven to eight animals in each experimental group). Statistically significant differences among treated, untreated, and control groups were tested using parametric Analysis of Variance (ANOVA). Post hoc tests (Tukey-Kramer (ANOVA)) were applied in situations where differences among groups were significant at the level of significance α = 0.05. After post hoc testing, the following significance levels were specified: extremely significant (*p* < 0.001), highly significant (*p* < 0.01), significant (*p* < 0.05), and not significant (*p* > 0.05). The untreated arthritis group was compared with healthy control animals (*), the treated arthritis groups were compared with untreated arthritic animals (^+^), and animals treated with a combination of FRA + MTX were compared to the MTX alone group (^#^).

## Figures and Tables

**Table 1:** Effect of FRA and MTX administered alone or in combination on basic parameters (change of body weight, change of hind paw volume, and arthritic score) in AA on the experimental days 14, 21 and 28.

Basic Parameters	Group				
	CO	AA	AA-MTX	AA-FRA	AA-FRA-MTX
Change of body weight (g)					
Day 14	68.6 ± 5.9	38.8 ± 6.0 *	67.3 ± 5.0 ^+^	36.2 ± 8.0	56.8 ± 6.4
Day 21	86.0 ± 7.0	31.0 ± 5.3 ***	52.4 ± 7.5	22.9 ± 4.6	64.2 ± 10.5 ^+^
Day 28	98.1 ± 8.3	48.1 ± 6.6 ***	51.1 ± 6.0	42.8 ± 4.3	67.0 ± 13.4
Change of hind paw volume (%)					
Day 14	8.3 ± 0.9	47.1 ± 6.4 ***	18.5 ± 5.2 ^++^	30.5 ± 8.7	13.1 ± 1.4 ^++^
Day 21	13.4 ± 2.0	80.2 ± 9.0 ***	44.9 ± 9.2 ^+^	65.0 ± 12.3	24.1 ± 7.8 ^+++^
Day 28	12.8 ± 1.8	60.7 ± 7.4 **	56.6 ± 11.2	50.3 ± 8.6	36.9 ± 10.5
Arthritic score (points)					
Day 14		16.3 ± 0.9	14.4 ± 0.7	13.6 ± 0.9	10.9 ± 0.6 ^+++/#^
Day 21		21.3 ± 1.2	19.3 ± 0.9	18.0 ± 1.3	14.1 ± 1.3 ^++/#^
Day 28		21.3 ± 1.1	20.5 ± 1.1	19.4 ± 1.2	15.9 ± 1.6 ^+^

CO, control group; AA, adjuvant arthritis group; AA-FRA, adjuvant arthritis group administered ferulaldehyde; AA-MTX, adjuvant arthritis group administered methotrexate; AA-FRA-MTX, adjuvant arthritis group administered ferulaldehyde and methotrexate. The data represent the mean ± SEM; *n* = 7–8. The symbols *, ^+^ and ^#^ show significant difference: * *p* < 0.05 versus CO; ** *p* < 0.01 vs. CO; *** *p* < 0.001 vs. CO; ^+^ *p* < 0.05 vs. AA; ^++^ *p* < 0.01 vs. AA; ^+++^ *p* < 0.001 vs. AA; ^#^ *p* < 0.05 vs. AA-MTX.

**Table 2:** Primer sequences.

Product	Sense Primer (5′-3′)	Antisense Primer (5′-3′)
IL-1β [22]	CCTCTGTGACTCGTGGGATG	GGGTGTGCCGTCTTTCATCA
β-actin	TCAAGATCATTGCTCCTCCTG	AGGGTGTAAAACGCAGCTCA
